# Plasmacytic markers as immunotherapeutic targets for primary CD20‐negative aggressive B‐cell lymphomas

**DOI:** 10.1111/bjh.70557

**Published:** 2026-05-21

**Authors:** Frank Neumann, Claudia Schormann, Onur Cetin, Igor A. Kos, Moritz Bewarder, Eva C. Schwarz, Stefan Lohse, Stephanie Maurer, Philipp B. Staber, Joerg T. Bittenbring, Sigrun Smola, Konstantinos Christofyllakis, Markus Hoth, Lorenz Thurner

**Affiliations:** ^1^ José Carreras Center for Immuno‐ and Gene Therapy and Department of Internal Medicine I Saarland University Medical School Homburg/Saar Germany; ^2^ Department of Oncology Bethanien Hospital Frankfurt am Main Germany; ^3^ Biophysics, Center for Integrative Physiology and Molecular Medicine, School of Medicine Saarland University Homburg Germany; ^4^ Institute of Virology Saarland University Homburg Germany

**Keywords:** ADCC, BCMA, CD19, CD20, CD38, CD79b, primary CD20‐negative aggressive B‐NHL, SLAMF7

To the Editor,

Aggressive B‐cell lymphomas (B‐NHLs) that lose classical B‐cell markers pose major therapeutic challenges. Among them, plasmablastic lymphoma (PBL), primary effusion lymphoma (PEL), KSHV/HHV8‐positive diffuse large B‐cell lymphoma (DLBCL) and anaplastic lymphoma kinase (ALK)‐positive large B‐cell lymphoma represent rare and often treatment‐refractory subtypes.^1^, ^2^, ^3^, ^4^ These entities are often associated with immune deficiency and characterized by downregulation of the transcription factors Paired Box Protein 5 (PAX5) and B‐Cell Lymphoma 6 (BCL‐6) and subsequent loss of CD19 and CD20 precluding the use of rituximab or other CD20‐ or CD19‐directed therapies. Morphologically and phenotypically, they share features of plasmacytic/blastic differentiation and thus commonly express CD38, Signaling Lymphocytic Activation Molecule Family Member 7 (SLAMF7) (CD319) and B‐cell maturation antigen (BCMA/CD269), caused by upregulation of Interferon Regulatory Factor 4 (IRF4) and B‐lymphocyte‐induced maturation protein 1 (BLIMP1).^5^ Targeting these plasmacytic features by bortezomib^6^, ^7^ or by the addition of CD38 antibodies has been studied in clinical case series and early clinical trials for PBL.^8^, ^9^ Furthermore, translational studies investigated SLAMF7 as target,^10^ but all these strategies do not represent a current established standard. The aim of the present study was to systematically examine the therapeutic potential of targeting plasmacytic antigens in primary CD20‐negative aggressive B‐NHL. We evaluated whether clinically approved immunotherapeutic antibodies for multiple myeloma—namely anti‐CD38 (daratumumab, isatuximab),^11^, ^12^ anti‐SLAMF7 (elotuzumab)^13^ or the bispecific BCMA×CD3 antibody (teclistamab)^14^—exert in vitro cytotoxic activity against CD20‐negative aggressive B‐cell lymphoma cell lines, with a focus on PEL, because the largest number of established cell lines is available for this subtype, but also limiting the transferability of the analyses to other primarily CD20‐negative entities. Furthermore, we explored whether pharmacologic activation of immune effector cells by lenalidomide or vitamin D supplementation could enhance antibody‐dependent cellular cytotoxicity (ADCC).

Four well‐characterized PEL cell lines (BC‐3, BCBL‐1, CRO‐AP2, CRO‐AP5) and control lines of CD20^+^ DLBCL and Burkitt lymphoma were analysed by flow cytometry for surface expression of CD19, CD20, CD79b, CD38, SLAMF7 and BCMA. The multiple myeloma cell line NCI‐H929 served as a positive control for BCMA expression. In vitro ADCC assays were performed using natural killer (NK) cells isolated untouched from healthy donors as effector cells. Target cells were preincubated with daratumumab, isatuximab or elotuzumab at concentrations ranging from 0.0001 to 1 μg/mL. To assess the impact of effector cell modulation, peripheral blood mononuclear cells (PBMCs) were treated in vitro with lenalidomide (1 μM, 24 h) prior to NK isolation, and in selected donors, vitamin D3 supplementation in vivo was performed to reach serum 25‐OH‐vitamin D3 levels ≥65 ng/mL.^15^ For BCMA × CD3 bispecific antibody assays, untouched isolated T cells or CD16^+^ NK cells from healthy donors were used as effectors at an effector‐to‐target ratio of 10:1 and 5:1.

Flow cytometry confirmed that all four PEL cell lines lacked CD20 expression, consistent with their plasmablastic origin. CD19 and CD79b were only very weakly expressed (Figure 1.1). In contrast, strong and uniform expression of CD38 and SLAMF7 and moderate but consistent BCMA expression were observed across all lines. Compared with the NCI‐H929 myeloma control, PEL cells expressed lower BCMA (CD269) surface levels (Figure S1), yet the presence of CD38, SLAMF7 and BCMA provided potential alternative immunotherapeutic targets. The very weak expression of CD19 correlates with the absence of significant NK‐cell activity under tafasitamab regardless of the dose and the PEL cell line (Figure 1.2). The same applies to CD79b and polatuzumab vedotin. A concentration of 1 μg/mL of polatuzumab vedotin significantly inhibited BCBL‐1 proliferation by 10%, compared to untreated cells (Figure 1.3), but this relatively weak inhibition only applied to BCBL‐1. Consistent with the weak expression of CD19 and CD79b, they are not promising targets for immunotherapy of PEL. Daratumumab and isatuximab induced dose‐dependent NK‐cell cytotoxicity proportional to CD38 surface density (Figure 1.4). Daratumumab was slightly more effective, reaching lysis rates of up to 15%–25% at 1 μg/mL, whereas slightly lower lysis rates were achieved with isatuximab at comparable concentrations (Figure 1.4, Figure S2.1–3). Elotuzumab‐induced NK‐cell activity varied. While less than 20% of BCL‐1, BC‐3 and CRO‐AP2 cells was lysed even at 1 μg/mL, this antibody induced lysis rates of up to 50% against CRO‐AP5 (Figure 1.5). This modest lysis in SLAMF7^+^ lines is consistent with its reliance on NK‐cell activation rather than direct ADCC (Figure 1.6). Although not strongly expressed on PEL cells, BCMA/CD269 was a somewhat more reliable target. Thus, the BCMA/CD3‐bispecific BiTE teclistamab induced a significant, T‐cell‐mediated ADCC of almost 25% against BCBL‐1 at a dose of 0.0001 μg/mL (Figure 1.7). Against BC‐3, the subjects' T cells lysed up to 17% of these PEL cells in a concentration‐dependent manner starting at 0.001 μg/mL teclistamab (Figure 1.6). Interestingly, despite its Fc‐silenced design, teclistamab induced comparable in vitro cytotoxicity when CD16^+^ NK cells were used as effectors, mirroring the T‐cell response pattern (Figure S3.1 & 3.2). The ADCC activity mediated by teclistamab in NK cells was significantly higher than that in T cells. This was evident not only in the higher target lysis by NK cells (45% vs. 25% for BCL‐1) but even more in the fact that these lysis rates were achieved at a significantly lower E/T ratio (4:1 vs. 10:1—compare Figure 1.6 & 1.7 vs. Figure S3.1 & 3.2). The level of BCMA (CD269) expression alone did not predict susceptibility, indicating that additional tumour‐intrinsic or effector‐cell factors influence the response. No cytotoxicity was observed against CD20‐negative lines when exposed to rituximab, confirming antibody specificity (Figure 1.4, Figure S2.1–3).

**FIGURE 1:**
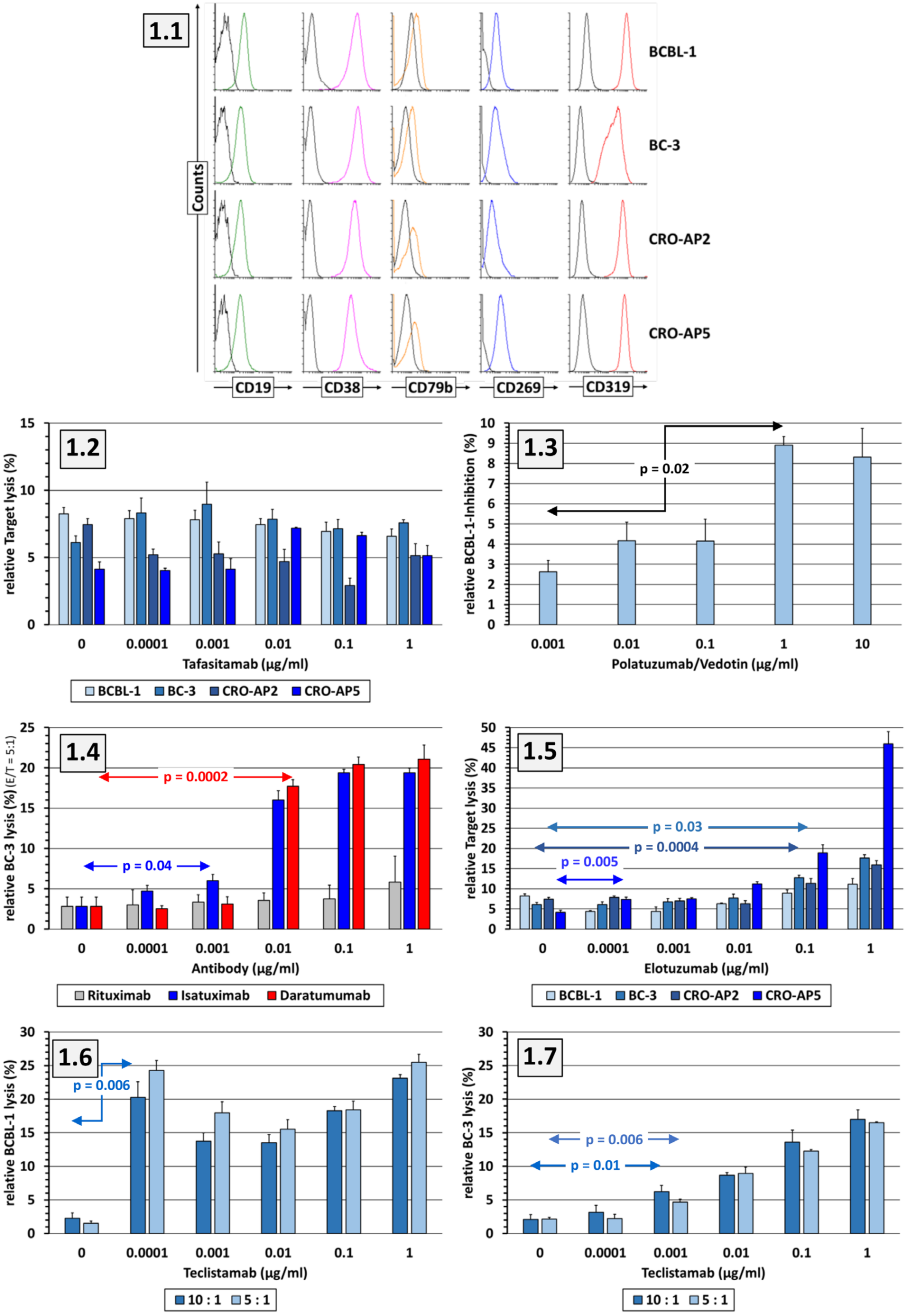
Cytotoxic potential of CD19, CD79b, CD269 and CD319 as primary effusion lymphoma (PEL) immune targets (1.1). Flow cytometric examination of the CD20‐negative cells from four lines of primary effusion lymphomas (BCBL‐1, BC‐3, CRO‐AP2 and CRO‐AP‐5) shows besides an extremely weak expression of CD79b, that another component of the B‐cell receptor, CD19, is also only moderately represented on the surface of the PEL cells. The same applies to the B‐cell maturation antigen (BCMA/CD269). In contrast, cyclic Adenosine diphosphate (ADP)‐ribose hydrolase (CD38) and Signaling Lymphocytic Activation Molecule Family Member 7 (SLAMF7) (CD319) are strongly expressed. The fluorescence signal of the respective antigen of the cells derived from the forward scatter (FSC)/side scatter (SSC)‐lymphocyte gate is shown. Isotype control appears in black. Natural killer (NK)‐cell‐mediated antibody‐dependent cellular cytotoxicity (ADCC) against the target cells of the four PEL lines under antibody treatment. (1.2) The CD19‐specific antibody tafasitamab was unable to induce significant ADCC. Only an antibody‐independent target lysis of around 5% was measured. (1.3) CD79b‐specific antibody/toxin conjugate polatuzumab/vedotin inhibits the proliferation of its target cells. Of the four PEL lines tested, only BCBL‐1 cells were inhibited significantly. However, this inhibition is therapeutically irrelevant with a reduced proliferation of less than 10%. (1.4) Unlike the previously described PEL surface markers, CD38 offers a better target for an antibody‐induced and NK‐cell‐driven ADCC. Against target cells of the PEL line CRO‐AP5 cells, daratumumab as well as isatuximab induced a significant ADCC starting at 0.001 μg/mL and 0.01 μg/mL respectively. However, native NK cells only lysed 10%–20% of these two PEL‐target cells. Daratumumab tended to induce a slightly stronger ADCC compared to isatuximab. Anti‐CD20‐specific rituximab was used as negative control indicating the antibody‐independent NK‐cell activity. (1.5) With the exception of BCBL‐1, the CD319‐specific antibody elotuzumab induced ADCC against the three other PELs tested. However, this was only significant against BC‐3 and CRO‐AP2 at an antibody concentration of 0.1 μg/mL and reached only 10%–15% of the maximum lysis. The cells of CRO‐AP5 showed the greatest elotuzumab sensitivity with a significant ADCC starting at 0.0001 μg/mL and higher lysis rates up to 45% of the maximum lysis at 1 μg/mL. Due to the weak expression of BCMA, the T‐cell response induced by the bispecific T‐cell engager teclistamab (anti‐BCMA/CD3) was not very pronounced. Against BCBL‐1 (1.6), 20%–25% of the target cells were lysed at just 0.0001 μg/mL teclistamab. However, the lysis rate did not increase at higher teclistamab concentrations. (1.7) Against cells of the Burkitt line BC‐3, a significant ADCC was measured starting at a teclistamab concentration of 0.001 μg/mL. This also increased with dose increases. However, the teclistamab‐induced lysis of BC‐3 cells remained rather modest at 17%.

A limitation of this study is that immune effector cells of healthy donors were utilized but not of patients, for reasons of availability. Patients with primary CD20‐negative aggressive lymphoma are regularly immunocompromised (iatrogenic, HIV, etc.) with quantitatively and functionally impaired immune effector cells. In general, R‐CHOP‐like therapies exert strong effects on immune effector cell counts and function in CD20‐positive DLBCL.^16^ However, the absolute immune effector cell counts were not the most important factor, but of subgroups as CD16^+^ T cells. These and the longitudinal dynamics have not been described so far for primary CD20‐negative aggressive B‐NHL, so under these circumstances, the effectivity of antibody‐dependent cytotoxicity either by NK or T cells remains speculative. Strategies to improve immune effector cell function beside the initiation of anti‐retroviral therapy in HIV‐positive patients or discontinuation of immunosuppressive therapies in patients with autoimmune history, a further strategy might be the use of immunomodulatory drugs (IMiDs) or even substitution of Vitamin D. Pretreatment of healthy donor PBMCs with lenalidomide substantially augmented ADCC in all lines (Figure 2). Mean cytotoxicity increased by approximately 175% compared with untreated NK cells, with a pronounced effect even at low antibody concentrations (0.01 μg/mL). Vitamin D supplementation in vivo (Table S1) provided an additional increase in lytic activity, between 25 and up to 100% (BCL‐1 and CRO‐AP2/daratumumab ≥0.01 μg/mL and CRO‐AP2/isatuximab ≥0.001 μg/mL—Figure 2), particularly in donors reaching 25‐OH‐vitamin D₃ levels >65 ng/mL. An almost equally strong enhancement was observed in BCL‐1, BC‐3 and CRO‐AP2, where lysis exceeded 80% at 1 μg/mL daratumumab. In contrast, CRO‐AP5 remained the least responsive PEL line despite measurable gains after both interventions.

**FIGURE 2:**
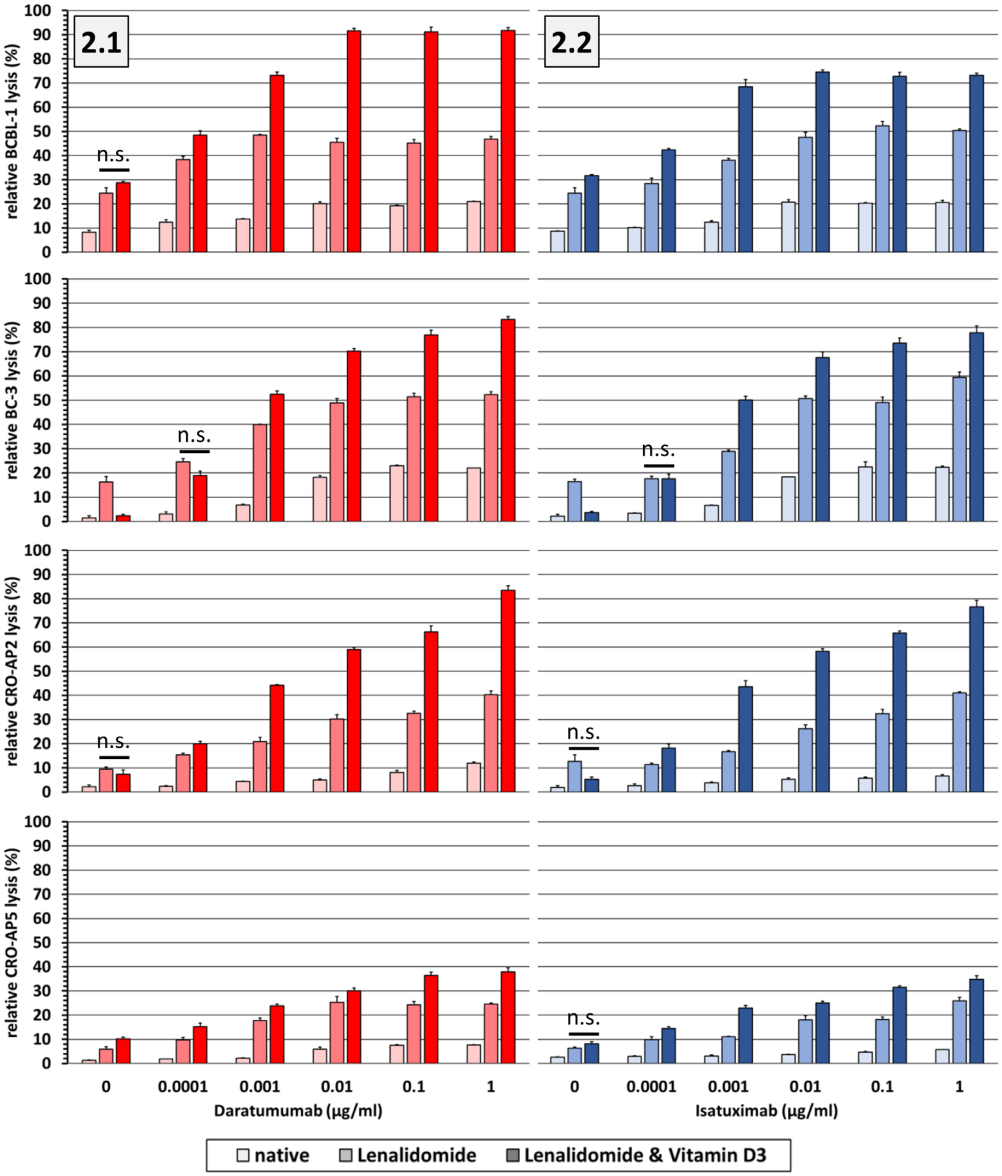
Boosting the CD38‐targeted antibody‐dependent cellular cytotoxicity (ADCC). After treatment of the peripheral blood mononuclear cell (PBMC) of subject 136 with lenalidomide and subsequent natural killer (NK)‐cell isolation, the ADCC activity (antibody ≥0.0001 μg/mL) was tested in a cytotoxicity assay against target cells of the four primary effusion lymphoma (PEL) lines labelled (2.1—red columns) with daratumumab and (2.2—blue columns) with isatuximab. Lenalidomide‐activated NK cells induced an ADCC that was two to four times stronger than the native NK cells, reaching lysis rates of 40% (CRO‐AP5) to 60% (BC‐3) at 0.1 μg/mL daratumumab. Isatuximab‐mediated ADCC after treatment of the NK cells with lenalidomide also increased significantly with lysis rates of just over 30% (CRO‐AP5) to more than 40%–45% (BCBL‐1, BC‐3, CRO‐AP2) compared to native NK cells. Thus, isatuximab‐mediated ADCC against PEL cells was significantly weaker overall compared to daratumumab. Additional supplementation of vitamin D (25‐OH D3 serum level from 23.3 to 65.6 ng/mL) in vivo before isolation of the NK cells of this volunteer increased the daratumumab‐mediated ADCC against the PEL target cells to 80% (BCBL‐1, BC‐3, CRO‐AP2), while under the same conditions, only approx. 50% of the CRO‐AP5 cells were killed. Compared to daratumumab, the target cells labelled with isatuximab did not benefit quite as much from the increased 25‐OH‐D3 serum level of the test person. Although the lysis rates (almost) always increased significantly after vitamin D3 supplementation, this was not to the same extent as against the PEL cells labelled with daratumumab. The exception was CRO‐AP2, in which the lysis rates almost doubled after vitamin D3 supplementation, regardless of whether it was loaded with daratumumab or isatuximab. The E/T ratio chosen for these ADCC assays was 5:1. If not explicitly marked as ‘not significant’ (n.s.), all differences between the individual lysis rates shown as columns were significant (*p* < 0.05). Light coloured columns = lysis rates of native cells, columns in medium brightness = after lenalidomide, dark coloured columns = after vitamin D3 and lenalidomide.

Our findings confirm that PEL express plasma‐cell–associated antigens that can be exploited by myeloma‐derived immunotherapies. Both CD38 and SLAMF7 are robustly expressed, while BCMA is detectable at lower but functionally relevant levels. The activity of anti‐CD38 antibodies in PEL seemed higher for daratumumab compared to isatuximab. Elotuzumab showed modest but measurable effects through NK‐cell engagement; however, we tested not the combination with lenalidomide for this antibody. The enhancement of ADCC by lenalidomide and, to a lower extent, by vitamin D supports prior observations of immune effector modulation and suggests clinically achievable strategies to improve antibody efficacy in these lymphoma subtypes. However, the previously described effect of vitamin D substitution on ADCC remains controversial, as in indolent lymphoma, the ILyAD trial did not report a benefit, and the results of the OPTIMAL > 60 trial regarding vitamin D substitution are awaited. The consistent in vitro activity of the BCMA × CD3 bispecific antibody further expands the spectrum of potential targets. While BCMA expression was lower in PEL than in myeloma, T‐cell–mediated lysis was still significant, implying that even moderate antigen density may suffice for therapeutic engagement. The observation that NK cells can also mediate BCMA‐directed cytotoxicity despite Fc silencing of teclistamab suggests secondary mechanisms of activation or residual Fc interactions. A limitation of this study is that G‐protein‐coupled receptor family C group 5 member D (GPRC5D), the target of another bispecific antibody approved for multiple myeloma, was not investigated.

From a translational perspective, our data indicate that PEL and hypothetically also other primary CD20‐negative aggressive B‐cell NHL entities might benefit from therapeutic strategies already approved for multiple myeloma. Our results show that anti‐CD38, anti‐SLAMF7 and BCMA × CD3 antibodies or BiTEs, respectively, alone or combined with lenalidomide or vitamin D exhibit strong in vitro cytotoxic activity against primary CD20‐negative aggressive B‐cell lymphomas.

The combination of anti‐CD38 antibodies with IMiDs and chemotherapy as CHO(E)P/EPOCH,^9^ or the use of BCMA‐directed bispecific antibodies, could represent rational approaches for evaluation in early‐phase clinical trials for patients with either therapy‐naïve or relapsed/refractory disease, as conventional B‐cell–directed immunotherapy fails. The findings establish a functional rationale for repurposing multiple myeloma immunotherapies in these rare, but high‐risk lymphoma subgroups, addressing a major unmet clinical need.

## AUTHOR CONTRIBUTIONS

**Moritz Bewarder:** Writing – review and editing; data curation. **Claudia Schormann:** Methodology; investigation; formal analysis; data curation; writing – review and editing. **Stefan Lohse:** Data curation; methodology; writing – review and editing. **Sigrun Smola:** Writing – review and editing; methodology. **Igor A. Kos:** Data curation; writing – review and editing. **Frank Neumann:** Conceptualization; methodology; formal analysis; validation; investigation; visualization; writing – original draft; writing – review and editing; data curation. **Joerg T. Bittenbring:** Writing – review and editing. **Philipp B. Staber:** Writing – review and editing; resources; funding acquisition. **Onur Cetin:** Writing – review and editing; data curation. **Eva C. Schwarz:** Data curation; writing – review and editing. **Markus Hoth:** Data curation; writing – review and editing; funding acquisition; conceptualization. **Stephanie Maurer:** Data curation; writing – review and editing. **Lorenz Thurner:** Conceptualization; methodology; formal analysis; visualization; writing – review and editing; writing – original draft; resources; funding acquisition. **Konstantinos Christofyllakis:** Writing – review and editing; data curation.

## FUNDING INFORMATION

Funding was provided by the Schwiete Foundation.

## Supporting information


Figure S1.
Figure S2.
Figure S3.
Table S1.

## Data Availability

Data are available upon request.
