# Supplementary material for: Plasmacytic markers as immunotherapeutic targets for primary CD20‐negative aggressive B‐cell lymphomas
Source: Br J Haematol. 2026 May 21;209(1):300–5. doi: 10.1111/bjh.70557 (PMC13340470; doi:10.1111/bjh.70557)
Supplement: Supplementary file 1 — Figure S1. Figure S2. Figure S3. Table S1. [file BJH-209-300-s001.pdf]

Plasmacytic Markers as Immunotherapeutic Targets for Primary CD20-negative Aggressive B-Cell Lymphomas

Frank Neumann et al.

Supporting Material and methods

Compliance with ethical standards

This study had been approved by the local ethical review board (Ethikkommission der Ärztekammer des Saarlandes – No. 178/17). All procedures performed in this study were in accordance with the ethical standards of this institutional committee and with the 1964 Helsinki declaration and its later amendments or comparable ethical standards._PBMC and NK cells were isolated from blood samples donated by voluntary staff members after written informed consent was obtained from all individual participants included in the study. These participants are listed in **Table S1** in supplemental data and material.

Cell lines and antibodies

Different cell lines from each lymphoma subtype were used as target cell in the ADCC assays. Of the DLBCL type, these were OCI-Ly3, TMD-8, and U2932. DAUDI and Ramos are HHV8+ Burkitt lymphomas and BCBL-1, BC-3, CRO-AP2, and CRO-AP5 are PEL subtypes. All cell lines were obtained from the DSMZ (Braunschweig, Germany). Cell lines were cultured at 37 °C and 5 % CO_2 and in supplemented cell medium according to the supplier's recommendations. Mycoplasma infection was excluded regularly by PCR. Cell lines were analyzed by flow cytometry for the expression of the different target antigens using fluorescent dye-coupled antibodies specific for CD19 (Beckman Coulter GmbH, Krefeld, Germany), CD20 (BioLegend, Amsterdam, The Netherlands), CD38, CD79b (BD Biosciences, Heidelberg, Germany), CD319/SLAMF7 (Miltenyi Biotec GmbH, Bergisch Gladbach, Germany) and BCMA (clone REA315/Vicky-1,

Miltenyi Biotec GmbH). Binding of the therapeutic antibodies isatuximab, daratumumab, elotuzumab, rituximab and teclistamab was also tested by flow cytometry using a fluorescent secondary anti-human Fc-antibody (DIANOVA GmbH, Hamburg, Germany). These Mab were obtained from the pharmacy of the Saarland University Medical Center and used at the indicated concentrations and incubated (37 °C) for 20 min with the corresponding target cells. FACS analyses were performed with a Navios 3L10C (Beckman Coulter GmbH) using the corresponding authorized software.

NK and T cells

PBMCs were isolated by density gradient centrifugation from the 50-100 ml EDTA blood donation of healthy donors. Thereafter NK cells were isolated from PBMCs by magnetic depletion of all non-NK cells using the CD56+/CD16+ human NK-Cell Isolation Kit (Miltenyi Biotech GmbH) according to the manufacturer`s instructions. The NK cells were either isolated one day before the ADCC assay and incubated overnight in wells coated with human serum albumin (HSA) without addition of cytokines. Alternatively, to investigate the effect of immunomodulatory imide drugs (IMiDs), PBMC were initially obtained and cultured in medium containing lenalidomide (1 µM) three days prior to the assay. In this case, the T helper cells are directly activated by lenalidomide, and their cytokines only then stimulate the NK cells. Afterwards, the activated NK cells were isolated as described before by depleting the non-NK cells in a magnetic field. In both cases, X-Vivo 15 media (Lonza, Basel, Switzerland) was used to which 10 % FSC had been added. The viability of the enriched NK cells was 99 % on average and the share of the CD16+ fraction was between 90 % and 98 %, as assessed by flow cytometry using the corresponding antibodies. The yield of CD16+ NK cells was between 3×10^6 cells and 1×10^7 cells. To further test for a possible improvement in ADCC due to the donor's vitamin D3 level, some donors had their vitamin D3 level raised to a mid-normal value of 65 ng/ml by

taking Cholecalciferol (Dekristol, Mibe GmbH, Brehna, Germany) and then their NK-cell activity was tested again.

Like the NK cells, the T cells were also isolated from the donor PBMCs by depletion of the non-T cells in the magnetic field on the day of the test, according to the provider's protocol (Pan T cell Isolation Kit, Miltenyi).

<u>ADCC assay</u>

NK/T cell-activity (against antibody-naïve target cells) and NK/T-cell-mediated ADCC (against antibody-treated target cells) were determined by the release of LDH from the respective target cells. NK/T-effector cells were co-incubated (96-well U-bottom, 4 h, 37 °C) with 4000 target cells using E/T-ratios of 10:1 or 5:1 (NK cells) or 4:1 and 2:1 (T cells), respectively. ADCC medium used to suspend the cells was X-Vivo 15 (Lonza) supplemented by 2% human serum albumin (HSA). The therapeutic antibodies to be tested were used at the concentrations indicated to treat the target cells immediately before the four-hour co-incubation. Thereafter supernatants were analyzed for their LDH content by ELISA using the Cytotoxicity Detection Kit Plus (Roche/Sigma-Aldrich Chemie GmbH) according to the manufacturer's instructions. ELISA plates were quantified using a Varioscan LUX™ type 3020 spectrophotometer and SkanIT 4.1™ software (Thermo Fisher Scientific, Darmstadt, Germany). NK-cell activity was calculated relatively to the detergence-induced maximum LDH release corrected by the spontaneous release of the corresponding target cells. Spontaneous LDH release averaged less than 5%. At values above 10%, the assay was discarded and repeated. Maximal and spontaneous lyses of the respective target cells were used as internal (inter-assay) controls.

<u>Statistical analysis</u>

NK/T-cell activity and ADCC assays were run with all samples in triplicates. The difference of the NK/T-cell activity against untreated targets (0 ng/ml of the corresponding antibody) and the antibody-treated targets was determined by *F-test* followed by a two-sided *Student's T-test*. ADCC activity at different 25-OH-D3 serum levels was compared using the *Wilcoxon signed-rank test*.

Supporting Table S1: Individual participants included in the study with special consideration of vitamin D3 serum levels in the context of enhancing NK cell activity.

Subject	Sex	Age	25-OH vitamin D3 serum level (ng/ml) before supplementation	after supplementation
**1**	male	58	23.6	87,5
**130**	female	29	28.8	n. d.
**135**	female	39	35.2	n. d.
**136**	female	25	23.3	65,6
**137**	male	31	n. d.	n. d.
**138**	female	31	29.6	74,8
**139**	male	27	n. d.	n. d.

Supporting Figures

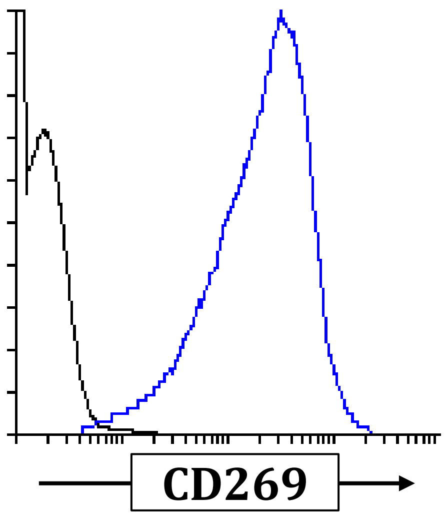

Supporting Figure S1: CD269 expression on myeloma cells

Cells of the myeloma cell line NCI H929 strongly express BCMA as it is typical for myeloma cells and thus forms the basis for successful therapy with teclistamab. The fluorescence signal of the respective antigen on the cells derived from the FSC/SSC-lymphocyte gate is shown. Isotype control appears in black.

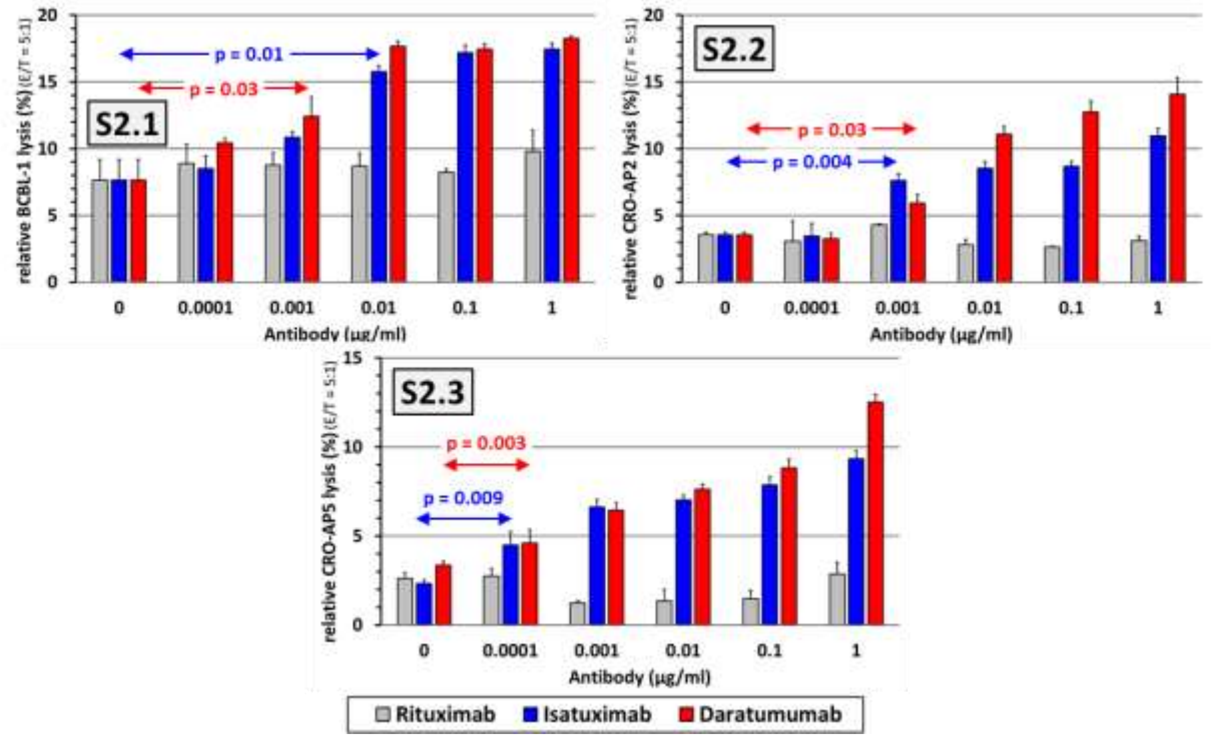

Supporting Figure S2: CD38 as a superior immune target on PEL

CD38 as target for the two antibodies daratumumab and isatuximab and thus offers greater immunotherapeutic potential for NK cell-mediated ADCC. As shown in individual diagrams BCBL-1 **(S2.1)**, CRO-AP2 (**S2.2)**, and CRO-AP5 (**S2.3)** both antiCD38 antibodies induced a significant dose-dependent ADCC starting at 0.0001 µg/ml (CRO-AP5, both antibodies), 0.001 µg/ml (CRO-AP2, both antibodies), 0.001 µg/ml (BCBL-1, daratumumab), and 0.01 µg/ml (BCBL-1, isatuximab), respectively compared to background activity without a dose-dependent chang for rituximab. P-values are only given for the lowest concentration level at which significant ADCC was present. For all higher antibody concentrations, ADCC was even more significant compared to untreated lymphoma cells, with correspondingly smaller p-values.

However, native NK cells only lysed 10-20 % of the target cells of the four PEL lines. Daratumumab tended to induce a slightly stronger ADCC compared to isatuximab. AntiCD20-specific rituximab was used as negative control indicating the antibody-independent NK-cell-activity against the corresponding cell line.

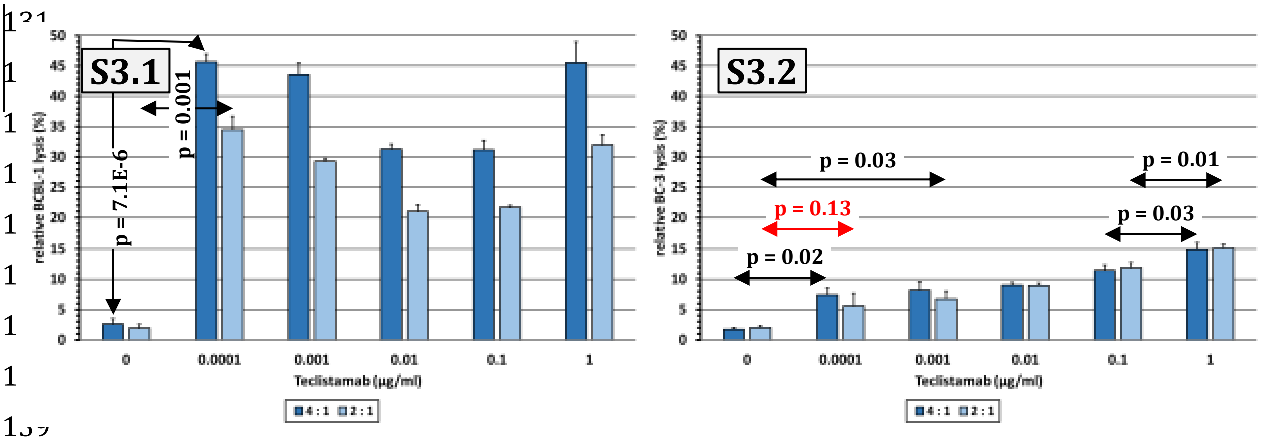

Supporting Figure S3: Teclistamab-mediated NK-cell-activity as an additional side effect against PEL

NK-dependent lysis of BCBL-1 **(S3.1)** or BC3 **(S3.2)** cells at two different E:T ratios in the absence or presence of different concentrations of **teclistamab**. NK cells also contribute to ADCC against PEL via the normally deactivated Fc portion of this CD269/CD3 bispecific antibody. Thus, NK cells induced a strong ADCC against BCBL-1 with teclistamab as low as 0.0001 µg/ml at both tested E/T ratios. The NK cell response against BC-3 cells was considerably weaker and only became significant at the low E/T ratio starting at 0.001 µg/ml antibody. Saturation was not observed up to 1 µg/ml. Using NK cells significantly higher lysis rates were achieved against BCBL-1 (**S3.1**) compared with T cells. Against BC-3 (**S3.2**), at a teclistamab concentration of 1 µg/ml only 15% of cells were lysed, just as with T cells, as shown in Figure 1F and G. However, this occurred with fewer NK cells per target cell (E/T = 4:1 [dark blue bars], E/T = 2:1 [light blue bars]).
